# Audience Response Systems and Missingness Trends: Using Interactive Polling Systems to Gather Sensitive Health Information From Youth

**DOI:** 10.2196/13798

**Published:** 2019-07-16

**Authors:** Tammy Toscos, Michelle Drouin, Mindy Flanagan, Maria Carpenter, Connie Kerrigan, Colleen Carpenter, Cameron Mere, Marcia Haaff

**Affiliations:** 1 Parkview Research Center Fort Wayne, IN United States; 2 Department of Psychology Purdue University Fort Wayne Fort Wayne, IN United States; 3 Lutheran Foundation Fort Wayne, IN United States

**Keywords:** mental health, youth, surveys and questionnaires, health care, software

## Abstract

**Background:**

The widespread availability and cost-effectiveness of new-wave software-based audience response systems (ARSs) have expanded the possibilities of collecting health data from hard-to-reach populations, including youth. However, with all survey methods, biases in the data may exist because of participant nonresponse.

**Objective:**

The aims of this study were to (1) examine the extent to which an ARS could be used to gather health information from youths within a large-group school setting and (2) examine individual- and survey-level response biases stemming from this Web-based data collection method.

**Methods:**

We used an ARS to deliver a mental health survey to 3418 youths in 4 high schools in the Midwestern United States. The survey contained demographic questions, depression, anxiety, and suicidality screeners, and questions about their use of offline resources (eg, parents, peers, and counselors) and Web-based resources (ie, telemental health technologies) when they faced stressful life situations. We then examined the response rates for each survey item, focusing on the individual- and survey-level characteristics that related to nonresponse.

**Results:**

Overall, 25.39% (868/3418) of youths answered all 38 survey questions; however, missingness analyses showed that there were some survey structure factors that led to higher rates of nonresponse (eg, questions at the end of survey, sensitive questions, and questions for which precise answers were difficult to provide). There were also some personal characteristics that were associated with nonresponse (eg, not identifying as either male or female, nonwhite ethnicity, and higher levels of depression). Specifically, a multivariate model showed that male students and students who reported their gender as other had significantly higher numbers of missed items compared with female students (*B*=.30 and *B*=.47, respectively, *P*<.001). Similarly, nonwhite race (*B*=.39, *P*<.001) and higher depression scores (*B*=.39, *P*<.001) were positively related to the number of missing survey responses.

**Conclusions:**

Although our methodology-focused study showed that it is possible to gather sensitive mental health data from youths in large groups using ARSs, we also suggest that these nonresponse patterns need to be considered and controlled for when using ARSs for gathering population health data.

## Introduction

### Background

Audience response systems (ARSs) are hardware- or software-based systems that allow presenters to interact with participants in real time, with audience members responding to questions posed by the presenter on handheld devices, and, in most cases, having their anonymous answers displayed on screen to the entire audience. Early iterations of ARSs relied on “clicker” hardware, a handheld device that had to be purchased by or distributed to audience members and used radio frequencies to send responses to the presenter’s computer-connected Universal Serial Bus (USB) drive. Owing to this hardware requirement, clicker systems were mainly marketed and adopted in higher educational contexts, where students would purchase the hardware and use it throughout the semester for quizzes and other class activities. However, the latest versions of ARSs (eg, Mentimeter and TurningPoint) are cloud software-based programs that allow for audience members to respond via their own connected devices, such as phones, tablets, or laptops. This transition from hardware- to software-based ARSs has increased the accessibility of these systems, expanding the possibilities of interactive education and real-time data gathering beyond traditional education environments. A notable feature of these ARSs is the confidentiality of audience members’ responses [1], a feature that might be particularly beneficial when gathering data or providing education on sensitive topics [2,3]. As audience members’ devices do not need to be registered (the survey is accessed via a weblink), participants can respond on provided devices or their own devices, without supplying any personal identifiers. In addition, unlike the older, hardware-based systems, the newest wave of ARSs neither have hardware costs associated with them nor do they have limits on the number of participants who can register their responses, which allows for large-scale, time- and cost-effective data gathering.

These innovations may be particularly useful to those in the health care industry, as it creates the potential to gather real-time health data on sensitive topics from a large number of participants. Indeed, there is some evidence that ARSs can be used successfully within the health care domain. In one of the first studies that employed this method, researchers used an ARS to provide information on anticoagulants to clinicians [4]. More recently, Tar-Ching et al [5] used an ARS to gather expert opinions on the barriers and priorities related to occupational health, and Toonstra et al [6] used an ARS to educate a diverse group of health care professionals on the safe rehabilitation of patients who were in intensive care units. Thus, ARSs have been used successfully for both educating health care professionals and gathering input from expert stakeholders. A study by Davis et al [7] showed that ARSs can also be used to simultaneously engage community members in health-related discussions and gather data during these health-related discussions. In their study, Davis et al [7] successfully used an ARS to educate community members and gather data about their knowledge of health disparities related to cancer.

Notably, most of the existing research on the use of ARSs within health care contexts has used adult samples, and only a few studies have focused on gathering health information from the youth. In their recent study, Gray et al [8] used an ARS to gather food intake and health (activity engagement) information from fourth- and fifth-grade children in the classrooms of 2 New York City schools. As compared with data gathered in paper-pencil surveys and data gathered 2 weeks later via the same ARS, the original data proved reliable. Meanwhile, MacGilleEathain [3] used an ARS to collect health data from secondary students in Scotland. In her study, adolescents responded to questions about sensitive topics (ie, sex and relationships), and she found the method “highly effective” (page 79) in gathering these types of data [3]. Combined, these studies suggest that ARSs can be employed successfully to gather health information within youth community settings. However, in both studies, students used clickers and not their own handheld devices, and both studies involved small groups of youths in classroom settings. Presently, there is no known research that has examined whether ARSs can be employed with youths using their own devices and in larger (nonclassroom) settings. In addition, although MacGilleEathain [3] suggested that ARSs might be used with the youth to gather data about suicidality, there is no known research that has examined the extent to which ARSs can be used successfully to gather data about sensitive mental health topics.

There is reason to believe that ARSs would be especially appealing to the youth, prompting high rates of survey response. The uptake of mobile technologies among adolescents (aged 13-17 years) in the United States is among the highest of any age cohort, with approximately two-third of the adolescents reporting that they have smartphone access (73%) and that they use social media (76%) [9]. In addition, because of their interactive features, ARSs have been lauded for their potential for audience engagement [1,4,10,11]. In support of this, a recent study comparing the effectiveness of ARSs with traditional hand raising in a classroom environment showed significant increases in student participation when using an ARS [10]. In addition, in an effort to improve end-of-semester course evaluation response rates, Turban et al [12] used an ARS and significantly improved rates from 55% with paper-based forms to 91% with ARS. Thus, the familiarity of the communication medium (ie, their own device) coupled with the anonymity and interactivity of the ARS may increase the likelihood that the youth will engage with an ARS-administered survey. However, some participants may not favor this response option. According to Almetria, Matusovich, and McCord [13], many college students prefer paper-and-pencil response options as opposed to electronic response options (eg, clickers or other software) for real-time experience surveys. In addition, Wyrick and Bond [14] found that as compared with paper-and-pencil surveys, sensitive questions delivered on the Web were 4 times more likely to be skipped by the middle and high school students in their sample. Consequently, as with other survey methods, a response bias may emerge in ARS surveys, whereby some individuals’ data are not included in parts of the study, for reasons that are not random [15].

Owing to the novelty of the method, it is currently unknown whether personal characteristics predict systematic nonresponse patterns for mental health surveys administered via ARS to the youth. However, in previous research with women who had undergone breast reconstruction after mastectomy, nonwhites and those from lower socioeconomic status were less likely to complete surveys [16]. In addition, Cheung et al [17] found that youths with more mental health issues were less likely to respond to voluntary survey questions than youths with fewer mental health issues, which resulted in a sample bias that skewed health behavior prevalence data. Thus, it is possible that the same types of personal characteristics that predict complete nonresponse (eg, race and mental health issues) might also predict missingness in Web-based survey data collected via ARS. Missingness on Web-based surveys might also be related to item placement, and declining response rates over the course of surveys have been noted with ARSs [18]. Whether from audience fatigue or a decline in the novelty effect, some participants who respond in the early parts of the survey may drop out, potentially leaving a nonrepresentative sample for later questions. According to Jääskeläinen and Lagerkvist [18], who tested ARS response rates among students with introductory physics tasks, these “small drops are unimportant.” However, when probing about sensitive mental health issues, this may not be true—respondents who drop out or choose not to respond over the course of a survey may be qualitatively different from those who complete the survey. This is the assertion that this study was designed to address.

### Objectives

In sum, the aim of this study was to examine the extent to which an ARS could be successfully employed to gather mental health information from youths in a large nonclassroom setting, using a software-based ARS that required students to use their own handheld devices. Our metrics for successful employment included an analysis of overall response rates, as well as a missingness analysis focused on decreases in responses through the course of the survey and nonresponse based on specific demographic and sociobehavioral sample characteristics (ie, age, gender, ethnicity, and depression and anxiety screen scores).

## Methods

### Youth Sample Recruitment

The goal of this study was to recruit an ethnically, racially, and economically diverse sample of high school students to participate in our ARS-delivered survey. To do this, we contacted school administrators in Northeast Indiana for possible participation. A total of 4 high schools, each in a different school district, with a total of 5156 students, agreed to participate. See Table 1 for enrollment data for the participating schools from the Indiana Department of Education [19].

**Table 1:** Demographic characteristics of students at 4 partnering high schools.

Characteristic	High school			
	A (N=1254), %	B (N=1641), %	C (N=1267), %	D (N=994), %
Female	50.32	47.41	45.86	47.5
Male	49.68	52.59	54.14	52.5
American Indian	<1.00	<1.00	<1.00	0
Asian	<1.00	<1.00	5.52	2.9
Black	<1.00	<1.00	29.67	29.0
Hispanic	6.54	2.50	16.02	9.1
Multiracial	1.44	2.19	7.26	5.1
Native Hawaiian or Other Pacific Islander	<1.00	0	0	<1.00
White	90.19	93.78	40.81	53.8
Free/reduced price meals	37.48	35.95	67.56	62.7

Each high school’s administration worked closely with our research team to plan and implement the consent process. As most high school students are minors (<18 years old), the consent process included communicating study details to parents or guardians. School administrators agreed to notify parents and guardians on our behalf through their standard means of communication. Schools used different methods to communicate with parents, including email, short message service text message, phone call, postal mail, printed paper copies sent home with students, or a combination of these. At schools for which standard communication modes included only electronic communication, printed paper forms were sent home with students for whom parental electronic communication was not established. Schools were required to communicate study details to parents at least one time and at least two weeks before the survey event date. All participating schools had existing protocols for the passive or “opt out” consent process, as they had used this method for other school-based activities. School administrators assumed responsibility for tracking those students who were not permitted to participate and providing them with an alternate activity (eg, time in the media room), using their standard procedures. On the day of the event, the high school principal was required to provide a signed, printed letter to the principal investigator, confirming that all parents had the opportunity to review the passive consent form and that all students whose parents opted out of the event had been accommodated with an alternative activity. Names of nonparticipating students and their parents were not shared with research team members to ensure their privacy. All procedures were approved by the research institution’s Institutional Review Board.

### Audience Response System Survey

A multidisciplinary team of health services researchers, nurses, informaticists, and suicide prevention experts developed an age-appropriate, 38-question mental health survey embedded within a video-based program focused on mental health needs and resources. The resulting product was a survey *event*, designed to be held in a large gymnasium or assembly hall, featuring an emcee (a local celebrity), a disc jockey playing popular music, and a series of prerecorded video clips featuring teen actors giving testimonials about common adolescent stressors, describing Web-based mental health resources for the youth, and asking the survey questions. During the live event, the youths were given time to respond to each question on their own handheld device, which they were instructed to bring at least one day before the event. A total of 2 schools had school-issued devices; the other 2 schools had tablets available for students who did not have a personal device or forgot to bring one to the event. The 1-hour “Tech to Stress” event was engineered by a contracted company that assisted with technological needs alongside study staff and managed video content production. The ARS comprised a proprietary polling software running on a Structured Query Language­-based platform on an individual server. An ad hoc network infrastructure was set up specifically for the event. The server was placed on a standalone Wi-Fi Protected Access-secured Wi-Fi network running dual band 802.11 N and G frequency standards. Multiple access points were deployed around the event area to maximize coverage, with extra care given to load balance the channels to avoid interference. A few of the schools turned off their regular wireless network transmissions in the area of the auditorium or gymnasium, as well as during the event, to help minimize interference. For added protection and security, the server was set on a separate subnet behind a network firewall to block access from those taking the survey from accessing the server directly and the data it contained. The students were not required to register their own devices in an identifiable way. The software assigned a random identifier to each device session in the event so that each individual’s survey responses were linked to 1 random identifier. At the end of the event, the data were then exported from the server to an encrypted USB drive and completely removed from the server. After each event, the ad hoc network was dismantled, and the server no longer held any event survey data. The Parkview Health Legal Department vetted the contractor and the contractor’s data security procedure. On the day of each school-wide event, students assembled into the auditorium or gymnasium, and they were given scripted instructions on how to register, connect to the polling technology, provide consent/assent, and complete demographic questions. Instructions were given on 2 video screens, and instructions were verbally given by the principal. If any of the participants had questions or technological difficulties, they were instructed to raise their hand so that study team members could assist them. Details about the “Tech to Stress Less” program are displayed in Table 2.

**Table 2:** Tech to Stress Less program components, description, and purpose.

Presentation step	Description	Purpose
Registration: 10 min (variable)	DJ^a^ was playing music throughout. Students assembled into the auditorium or gymnasium, and they were given scripted instructions on how to register, connect to the polling technology, provide consent, and complete demographic questions. Instructions were given on 2 video screens, and instructed were verbally given by the principal. If any of the participants had questions or technological difficulties, they were instructed to raise their hand for assistance.	Obtain Consent; Provide instructions for polling procedure
Welcome: 2.5 min (variable)	DJ was playing music softly in the background throughout. Students were welcomed by a live speaker, using prescripted verbiage, who kicked off the event, followed by a high-energy musical performance, with music played by a DJ.	Promote participant engagement
Stress content: 20.75 min	Video content alternated with survey questions that participants answered with a personal device (laptop, tablet, and mobile phone). This section comprised prerecorded educational videos on stress (6.18 min) and testimonial videos of the youth talking about their stress (5.57 min), intertwined with survey questions introduced through video (9.00 min).	Presentation of adolescent stress and mental health concerns; Assess prevalence of mental health concerns
Tech content: 22.22 min	Video content alternated with survey questions that participants answered with a personal device (laptop, tablet, and mobile phone). This section comprised prerecorded informational videos on existing technologies (6.22 min), intertwined with survey questions introduced through video (16.00 min).	Educate about TMH^b^; Obtain youth ratings of TMH
Conclusion+final question: 2.5 min (variable)	Youth rated satisfaction with the event (0 to 10 scale)	Obtain ratings of event; Dismissal

^a^DJ: disc jockey.

^b^TMH: telemental health technologies.

### Measures

In total, students were presented with 38 questions during this event. At the outset, students responded to 7 items to assess demographics (age, gender, and race), anxiety, and depression. *Depression and Anxiety* were measured using the Patient Health Questionnaire (PHQ)-4 [20], a validated, ultrabrief measure of depression and anxiety [20-22] that has been found to be a valid tool in the mass screening of young adults [23]. Students responded on a 4-point Likert scale (0=*not at all*, 3=*nearly every day*) about how often in the last 2 weeks they had experienced depression and anxiety symptoms. We computed scores for the subscales (depression Cronbach alpha=.76; anxiety Cronbach alpha=.82). According to the scale parameters, subscale scores from 0 to 2 are classified as normal to mild, and scores from 3 to 6 are classified as moderate to severe in their symptomology. The remaining items assessed previous mental health provider visits, suicidality (Youth Risk Behavior Surveillance System [24]; YRBSS_1 and YRBSS_2), stress, coping strategies, preferred telemental health tool features, use of telemental health tools, openness to using telemental health tools, comfort with face-to-face therapy, and satisfaction with the event. All items were closed ended. The order of questions was varied so that respondent fatigue would not unduly affect particular (later) questions. Specifically, for questions 15 to 30, the order of presentation was varied such that, for 2 schools, questions 25 to 30 were presented earlier (as questions 15 to 20), and questions 15 to 24 were presented later (as questions 21 to 30). Therefore, the question numbers listed below refer to the order in which the item was presented. However, when content was analyzed (eg, desire for anonymity), comparisons were made across different question numbers. All items are in Multimedia Appendix 1.

### Data Analysis

All data were aggregated by research study personnel. Data analyses from the larger study are presented elsewhere (Toscos et al, in press). For this study, which was focused on the nuanced analysis of the ARS response rates, descriptive statistics were calculated for demographic, anxiety, and depression. PHQ scores for anxiety and depression items were dichotomized such that scores 3 and higher represented moderate-to-severe levels. Plots were constructed to show the overall percentage of missing responses per question. To examine missingness in these survey data, a count variable was created to represent the total number of missing responses for each participant. Here, a missing value was either a skipped response or, where available as an option, a “Prefer not to answer” response. In both cases, the student’s answer was coded as missing, whether it was from intentional or unintentional avoidance of answering the question. In total, 38 items comprised this count variable. A second count variable was computed to exclude the final 4 items to account for the technology issues that may have impacted response rates. Moving averages were calculated for percentage of missing responses for each set of 4 sequential questions. Moving averages were compared with individual item response rates to identify questions with higher missingness than items immediately preceding and following. In addition, as per Cameron and Trivedi [25], a generalized linear model using a negative binomial distribution was tested because of the use of count data and overdispersion. In this model, demographics, PHQ anxiety score, and PHQ depression score were entered as predicting number of missed responses. In post hoc tests, all pairwise comparisons among schools for number of missing items were conducted using Tukey adjustment for multiple comparisons. Analyses were conducted using IBM SPSS Statistics Version 24 (IBM Corp.) and SAS software 9.4. Copyright 2014 SAS Institute Inc. SAS and all other SAS Institute Inc product or service names are registered trademarks or trademarks of SAS Institute Inc, Cary, NC, USA.

## Results

**Figure 1:**
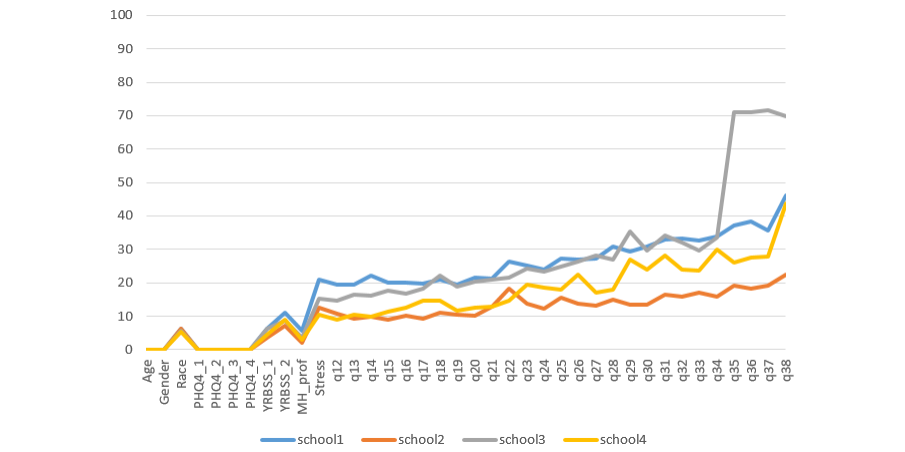
High school survey nonresponse: percentage of missing responses by school (N=3418). PHQ: Patient Health Questionnaire; YRBSS: Youth Risk Behavior Surveillance System; MH-Prof: visit with Mental Health Professional.

**Figure 2:**
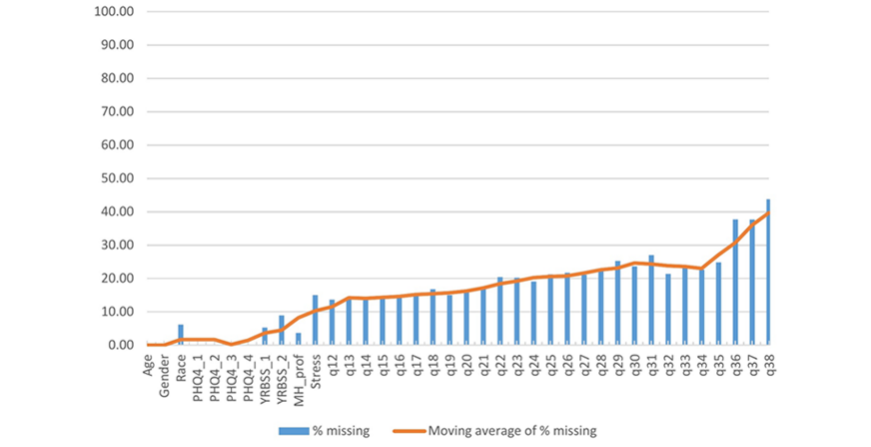
Percentage of missing responses and moving average of missed response percentage by item (N=3418). School 3 missing items omitted from q35 to q38 because of technology issue. PHQ: Patient Health Questionnaire; YRBSS: Youth Risk Behavior Surveillance System; MH-Prof: visit with Mental Health Professional.

In total, 3418 high school students participated in the survey events. Of these, 49.56% (1694/3418) of the high school students were female, 46.84% (1601/3418) of the high school students were male, and 3.60% (123/3418) of the high school students responded “other” to gender. Mean age was 16.12 years (SD 1.22, range 13-19), and ethnicities were non-Hispanic white (60.47%, 2067/3418), black (12.96%, 443/3418), Latino (7.90%, 270/3418), Asian (2.43%, 83/3418), Native American (1.64%, 56/3418), South Asian or Indian American (0.76%, 26/3418), Middle Eastern (0.80%, 27/3418), and other or multiracial (6.85%, 234/3418); 6.03% (206/3418) of the high school students selected “prefer not to answer,” and 0.18% (6/3418) did not respond. The average PHQ depression score was 1.39 (SD 1.40), and average PHQ anxiety score was 1.63 (SD 1.51), with 23.71% (809/3412) and 30.80% (1051/3412) of the high school students meeting the minimum score for moderate-to-severe depression and anxiety, respectively. As shown in Figure 1, the number of missing responses steadily increased over the course of the survey event. To illustrate, overall missingness for Q12 was 13.63% (466/3418), overall missingness for Q23 was 20.28% (693/3418), and overall missingness for Q33 was 23.20% (793/3418). For the final item (satisfaction rating for the event) at schools 1, 2, and 4, 35.06% (905/2581) of the high school students did not respond. At school 3, percentage of missed responses was high for the final 4 items (71.09%, 595/837; 70.97%, 594/837; 71.57%, 599/837; 69.89%, 585/837) because of a technology issue. In terms of participants’ nonresponse for *specific items*, there were jumps in missingness (on the basis of difference between item moving average response rate and actual response rate, Figure 2) for the items assessing race, previous suicidality (YRBSS_2 ), stress, preference for learning to manage stress (q22 for schools 1, 2; q29 for schools 3, 4), and preference for anonymity if discussing problems on the Web (q26 for schools 1, 2; q31 for schools 3, 4). For these latter 2 items with high missingness, the number of missing responses was higher when the item was administered at a later point in the survey. The average number of total missed responses was 5.61 (range 0-32, SD 8.41, and n=2575), omitting school 3. Including school 3 and omitting the final 4 items involved with technology issues, total missed responses was 4.80 (range 0-33, SD 7.36). Of all participants, 86.92% (2971/3418) responded to 20 or more items, and 25.39% (868/3418) of the participants responded to all items. However, 13.08 % (447/3418) of the participants responded to 19 or fewer items. In a multivariate model, race, gender, school, and depression were significantly related to number of missed items, whereas age and anxiety were not related to number of missed items (see Table 3). Male students and students reporting “other” gender had significantly higher numbers of missed items compared with female students. Similarly, nonwhite race and higher depression score were positively related to the number of missing survey responses.

**Table 3:** Negative binomial generalized linear model results for combined demographics and depression score predicting number of missing items on high school survey (N=3206).

Predictor	*B* ^a^	SE^b^	Wald 95% confidence limits	*P* value
Intercept	0.86	0.10	0.67, 1.04	<.001
Race (not white)	0.38	0.07	0.25, 0.52	<.001
Gender (male vs female)	0.32	0.06	0.19, 0.44	<.001
Gender (other vs female)	0.50	0.19	0.12, 0.87	.009
School (1 vs 4)	0.56	0.10	0.37, 0.75	<.001
School (2 vs 4)	–0.10	0.09	–0.29, 0.08	.27
School (3 vs 4)	0.33	0.09	0.15, 0.51	<.001
Patient Health Questionnaire depression	0.06	0.02	0.02, 0.11	.004

^a^*B*: unstandardized parameter estimate.

^b^SE: standard error.

**Figure 3:**
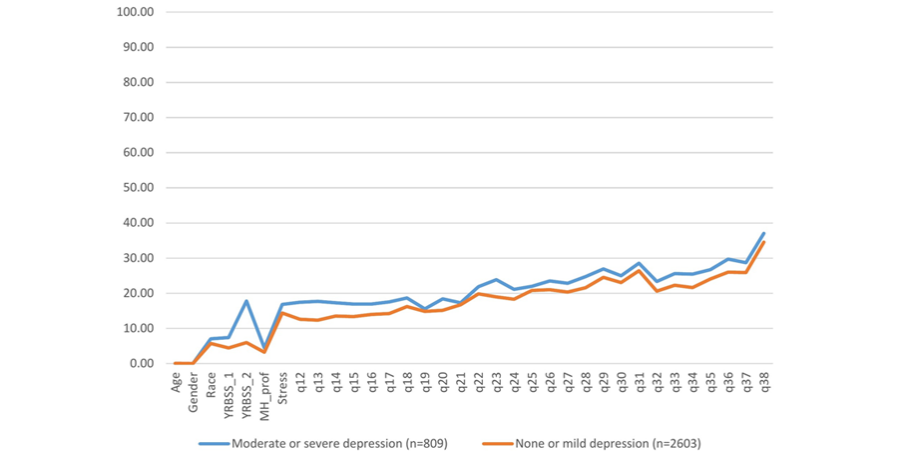
High school survey nonresponse: percentage of missing responses by item for depressed and nondepressed students (N=3412). School 3 missing items omitted from q35 to q38 because of technology issue. YRBSS: Youth Risk Behavior Surveillance System; MH-Prof: visit with Mental Health Professional.

As shown in Figure 3, the pattern of missed responses for depressed students was, although at a higher percentage, largely similar to nondepressed students, with a striking difference for the item assessing previous suicidality (YRBSS_2). In post hoc comparisons among schools on missingness, 4 of 6 comparisons were significant (school 1 vs 2: *P*<.001; school 1 vs 4: *P*<.001; school 2 vs 3: *P*<.001; school 3 vs 4: *P*=.002). However, school 1 and school 3 (*P*=.05) did not differ from one another, and school 2 and school 4 did not differ from one another (*P*=.69).

## Discussion

### Principal Findings

A software-based ARS was successfully employed to gather mental health information from youths in a large nonclassroom setting at 4 high schools. Overall, 80% of youths responded to more than half of the survey items, and one-fourth of the youth responded to all items. This is encouraging, as it seems to be particularly challenging to gather health data from the youth. For example, in a previous study, a mailed survey to adolescents about their health needs (including depressive symptoms) yielded a 33% response rate, with only a modest improvement in response rate when phone call reminders were conducted as an additional strategy to enhance response rates [26]. Although response rate was not measured in this study (our missingness analyses included only students who logged into the system at least once), we were able to gather health data from a wide variety of youths with different socioeconomic and mental health backgrounds. There is some evidence that this can also be accomplished with paper-and-pencil surveys. The 2015 Youth Risk Behavior Surveillance survey, for example, which was administered in classrooms to students at 125 public and private high schools, boasted an 86% response rate [27]. The advantage of the ARS approach to data gathering over these paper-and-pencil school-based collection methods is cost. Traditional research may have high costs (either financial or time or both) associated with the materials and data entry for mailed surveys, multiple waves of reminders and solicitation for electronic surveys, and outreach required to obtain large sample sizes [28]. Newer ARSs (eg, Mentimeter) have low annual fees associated with them (eg, US $100 or less), and students can connect via school (or portable) Wi-Fi or a cellular data connection. Thus, our sampling approach (surveying students en masse in a school setting) coupled with the ARS may provide a quick, relatively inexpensive data collection process for gathering data from the youth. Importantly, adolescents reported on sensitive mental health topics, demonstrating that ARS is a possible methodology for assessing these subjects. This aligns with previous research demonstrating that the privacy permitted by self-administered surveys facilitates disclosure of depressive symptoms and other nondesirable behaviors [29,30]. However, previous research has also shown that item response tends to decline over the course of ARS-delivered surveys [18], and item missingness tends to be higher for items that are too personal or intrusive [30,31]. Both of these trends held true for our sample. With regard to declining response rates, missingness increased steadily over the course of the survey: the percentage of students who skipped responses increased from a high of 17% for a question in the first half of the survey to a high of 35% for the last question of the survey. These findings emphasize the importance of item placement; critical survey items should be placed at the start of surveys when using ARSs to gather data from the youth. Additional analyses showed that some types of questions were skipped more than others, and examining the pattern of skipped responses revealed several themes. First, more sensitive questions (eg, about race, suicidality, and stress) were more likely to be skipped than the questions immediately preceding those questions, regardless of their place in the survey. This aligns with findings from Asgeirsdottir et al [30], who found, using their paper-pencil survey methods in Swedish high schools, that 10.8% of high school teens skipped questions about sexual abuse as opposed to less than 5% who skipped questions on other, less sensitive, topics (eg, family conflict, depressed mood, and anger). Kays et al [31] also found that college students were less likely to answer sensitive questions than nonsensitive questions; however, they also found that respondents were more likely to respond to sensitive questions via Web-based surveys than via the paper responses. Other spikes in nonresponse may have been because of the question requiring more cognitive effort or being a poorly constructed item (eg, preference for learning to manage stress), which resulted in more missing data when this item was administered later in the survey. Live survey administrators may want to consider giving additional prompts throughout the survey (eg, reassuring participants of their anonymity throughout or emphasizing the importance of completing sensitive questions) and increasing the specificity and relevance of items so that these spikes in missingness are minimized. The impact of adding these or other prompts on participants’ response rates during live ARS surveys is a direction for future research. On an individual level, amount of missing data was related to specific demographic and sociobehavioral characteristics (ie, gender, ethnicity, and depression scores). Previous studies of nonresponse have found more nonresponse for blacks and Hispanics than whites [32,33]. This trend was also evident in our sample—on average, nonwhite students (including black and Hispanic students) skipped 17% of the items, whereas white students skipped only 11% of the items. Nonresponse rates were also related to gender: “other” selection for gender was related to the highest rates of nonresponse (27%). This finding is consistent with previous studies that have shown that males have greater levels of nonresponse than females [34,35]. Finally, depression level was related to nonresponse in our sample. A previous diary study showed that nonresponse was related to depressive symptoms, such that depressed individuals had lower compliance with multiple survey completions per day [35]. In addition, in a survey on alcohol consumption, excessive drinkers were less likely to respond to the survey [36]. Combined, these previous studies suggest that those with behaviors or symptoms that fit the stigmatized topic of interest may be less likely to respond to survey items. This was also true in our anonymous survey delivered over ARS—the interactive polling system and group survey approach did not appear to overcome these issues. With consideration for all of the individual characteristics that affected item response, statistical analyses of live survey response data need to account for these patterns of nonresponse and employ statistical corrections.

### Limitations

Several limitations must be noted. First, we have limitations in the sample. Specifically, the sample included only adolescents enrolled in school, excluding home-schooled adolescents and adolescents who have dropped out, as well as students who were not present on the day the survey was administered. As previous researchers have suggested, both adolescents not enrolled in school and absent could differ from those present for the survey event [37]. In addition, some students experienced technological issues that precluded them from participating on individual questions and may have pushed them out of the survey, forcing the start of a new survey instance for some participants. For example, some students in school 1 experienced difficulty entering and remaining connected to the local Wi-Fi network, and some students in school 3 experienced technological issues that prevented them from answering the final 4 questions. These sampling issues, in addition to other malfunction issues that could have occurred without our knowledge (eg, low battery power in devices or devices freezing during survey participation), limit the generalizability of our findings. However, members of the research team noted that the technological issues appeared to be minimized in schools where students accessed the ARS survey with school-issued devices. As bring your own device (BYOD) programs gain popularity [38], researchers should explore the extent to which ARS survey responsiveness is related to whether participants use their own devices or school- or company-issued devices. It may be that those in underserved populations, who may have the greatest mental health needs, may also be the ones who do not have their own devices, prohibiting their response in a BYOD environment. This is an important issue to explore in future research. Second, some items used in the survey were researcher developed and not previously validated. As a result, item clarity or acceptability could have influenced nonresponse rates. For example, the item “How would you most prefer to learn to manage your stress” had the response options “Learn about it in school; Ask a healthcare professional; Use as an app or website; Find other ways; Prefer not to answer.” This item requires a respondent to project one’s self into a hypothetical situation and select a most likely behavior from a list of predefined options. However, it is possible that all of these options were equally unappealing to the student, and as that was not an option provided, the student chose not to answer. Moreover, participants’ selection of “other” gender did not clearly indicate gender identity for these students. Questions that more accurately assess gender—such as “What is your current gender identity?” and “What gender were you assigned at birth?”—as have been used in previous research [39], could be incorporated in future studies to explore this issue of gender and survey nonresponse more directly. Third, differences among schools (ie, behavioral norms, teaching atmosphere, and rural vs urban location) or survey administration events (ie, day of the week, time of year) were not considered in this research project. These unmeasured factors likely exerted some influence on missingness, as school was a significant predictor for rate of missing responses. Future research should consider environmental or contextual issues that could impact survey response rates. Fourth, it may be that some of the most vulnerable students in terms of mental health needs were not responding to survey items (either skipping or selecting “prefer not to answer”), which may bias the statistics so that the group appears more psychologically healthy than it really is. Although the anonymity of the system and ease of responding may have encouraged response for some of these individuals, the ARS does not completely eliminate this type of response bias. Future iterations of ARS research with adolescents should test ways to increase response rates in these populations, perhaps by offering these individuals more assurances of the anonymity of their data or providing other participation incentives. Finally, it is possible that the entertainment-based presentation might have motivated students to respond, which might have contributed to the high response rate. Alternatively, the videos and entertainment activities in a large-group setting may have had an opposite effect on some students, increasing rates of nonresponse or even promoting a social desirability bias in student responses, a risk that jeopardizes the validity of the main findings of the larger study. Thus, a limitation of this study is that we did not specifically ask students questions about the ARS and whether they felt the system was truly anonymous, whether our methods for data collection were engaging, or whether they felt that other methods of data gathering (eg, paper-pencil surveys or ARS in the classroom) would lead to a greater number of (or less biased) responses. Future research examining data gathering systems should focus on these points more directly. In addition, we did not ask or measure the extent to which students learned about Web-based resources for mental health (ie, content-related knowledge) from the process of participating in the ARS-delivered survey. These clarifying questions would provide helpful feedback about the best ways to introduce and use ARSs and which types of settings work best for which type of students. This is a valuable direction for future research.

### Conclusions

Despite these limitations, this study demonstrated that an ARS can be used to gather sensitive mental health information from the youth, and we assert that these findings may be generalizable to different topical interests and community settings. Future research should investigate whether ARSs can be employed successfully to gather data on other health topics from adolescent samples. As software-based ARSs are beginning to emerge as cost-effective data gathering solutions outside of traditional education and business environments, there is great opportunity to further develop the methodology and data collection procedures for gathering health information from adolescents, as well as adult participants, in different types of community settings.

## Appendix

Multimedia Appendix 1 Survey items and response set.

## Abbreviations

ARS: audience response system
BYOD: bring your own device
PHQ: Patient Health Questionnaire
USB: Universal Serial Bus
YRBSS: Youth Risk Behavior Surveillance System
