# Supplementary material for: Audience Response Systems and Missingness Trends: Using Interactive Polling Systems to Gather Sensitive Health Information From Youth
Source: JMIR Form Res. 2019 Jul 16;3(3):e13798. doi: 10.2196/13798 (PMC6664663; doi:10.2196/13798)
Supplement: Multimedia Appendix 1 [file formative_v3i3e13798_app1.pdf]

Question (alternate)	Question	Response Set
Age	What is your age?	13 - 19
Gender	I identify my gender as:	A. Male<br>B. Female<br>C. Other<br>D. Prefer not to answer
Race	What is your race/ethnicity?	A. Non-Hispanic White or Euro-American<br>B. Black, Afro-Caribbean, or African American<br>C. Latino or Hispanic American<br>D. Native American or Alaskan Native<br>E. East Asian or Asian American<br>F. South Asian or Indian American<br>G. Middle Eastern or Arab American<br>H. Other or Multi-racial<br>I. Prefer not to answer
PHQ4_1	Over the last 2 weeks, how often have you been bothered by the following problems?<br>• Feeling nervous, anxious or on edge	A. Not at all<br>B. Several days<br>C. More than half the days<br>D. Nearly every day<br>E. Prefer not to answer
PHQ4_2	Over the last 2 weeks, how often have you been bothered by the following problems?<br>• Not being able to stop or control worrying	A. Not at all<br>B. Several days<br>C. More than half the days<br>D. Nearly every day<br>E. Prefer not to answer
PHQ4_3	Over the last 2 weeks, how often have you been bothered by the following problems?<br>• Little interest or pleasure in doing things	A. Not at all<br>B. Several days<br>C. More than half the days<br>D. Nearly every day<br>E. Prefer not to answer
PHQ4_4	Over the last 2 weeks, how often have you been bothered by the following problems?<br>• Feeling down, depressed, or hopeless	A. Not at all<br>B. Several days<br>C. More than half the days<br>D. Nearly every day<br>E. Prefer not to answer
YRBSS_1	During the past 12 months, did you ever feel so sad or hopeless almost every day for two weeks or more in a row that you stopped doing some usual activities?	A. Yes<br>B. No<br>C. Prefer not to answer
YRBSS_2	During the past 12 months, did you ever seriously consider attempting suicide?	A. Yes<br>B. No<br>C. Prefer not to answer
MH_Prof	Have you ever been to a therapist for a counseling session?	A. Yes<br>B. No

		C. Prefer not to answer
Stress	On a scale of 0 to 10, where 0 means you have "no stress" and 10 means you have "a great deal of stress", how would you rate your average level of stress during the past month?	0 - No stress, 10 - A great deal of stress
Q12	When you experience a high level of stress, do you prefer to:	A. Deal with it on your own<br>B. Reach out for support from others<br>C. Reach out to a healthcare professional<br>D. It depends on the situatio<br>E. Prefer not to answer
Q13	With whom can you openly and honestly discuss your stress/problems? (please select all that apply)	A. Parent/guardian<br>B. Other family member<br>C. Friend/peer<br>D. Teacher, guidance counselor, other school staff<br>E. Health professional<br>F. Other adult<br>G. Someone else, none of the above<br>H. No one<br>I. Prefer not to answer
Q14	When you need to talk to someone about your stress or problems, how do you prefer to talk to them?	A. In person, face-to-face<br>B. Phone call<br>C. Text or online chat<br>D. Video chat (Skype, FaceTime, etc.)<br>E. Social media (Instagram, SnapChat, Facebook, etc.)<br>F. Other<br>G. Prefer not to answer
Q15	Would you prefer to share your stress or problems with people who have the same stress or problems as you?	A. Yes, definitely<br>B. Yes, but it depends on the situation or how I'm feeling<br>C. Maybe, I don't know<br>D. No, not necessarily<br>E. No, definitely not<br>F. Prefer not to answer
Q16	Have you ever experienced people supporting you on social media through challenges or tough times?	A. Yes, frequently<br>B. Yes, occasionally<br>C. No<br>D. Prefer not to answer
Q17	If you wanted to talk to a counselor or therapist about your stress or problems, do you think your parents would be willing to help you get an appointment with one?	A. Yes, definitely<br>B. Yes, probably<br>C. Maybe, I don't know<br>D. No, probably not<br>E. No, definitely not<br>F. Prefer not to answer
Q18	If you wanted to talk to a counselor or therapist about your stress or problems, how comfortable would you	A. Very comfortable<br>B. Comfortable<br>C. Not sure

	be asking your parents to help you get an appointment with one?	D. Uncomfortable<br>E. Very uncomfortable<br>F. Prefer not to answer
Q19	How comfortable would you be talking to a counselor or therapist face-to-face in real life?	A. Very comfortable<br>B. Comfortable<br>C. Not sure<br>D. Uncomfortable<br>E. Very uncomfortable<br>F. Prefer not to answer
Q20 (Q27)	Have you ever used an app or website designed to help you manage your stress or feelings on your own, like the examples shown?	A. Yes<br>B. No<br>C. Maybe, I don't know<br>D. Prefer not to answer
Q21 (Q28)	Would you ever use an app or website designed to help you manage your stress or feelings on your own, like the examples shown?	A. Yes, definitely<br>B. Yes, but it depends on the situation or how I'm feeling<br>C. Maybe, I don't know<br>D. No, not necessarily<br>E. No, definitely not<br>F. Prefer not to answer
Q22 (Q29)	How would you most prefer to learn how to manage your stress or feelings? Would you rather…	A. Learn about it in school<br>B. Ask a healthcare professional<br>C. Use an app or website, like the examples shown<br>D. Find other ways<br>E. Prefer not to answer
Q23 (Q26)	Would you rather use an app or website to help you manage your stress…	A. On your own<br>B. With the guidance of a healthcare professional<br>C. Not use an app or website to manage your stress at all<br>D. Prefer not to answer
Q24 (Q30)	Would you rather express your feelings and reduce stress by creating artwork…	A. On your own and keeping it to yourself<br>B. On your own and sharing it with a few people<br>C. To share it in an online expressive arts therapy group<br>D. Not creating artwork at all<br>E. Prefer not to answer
Q25 (Q31)	If you were going to discuss your stress or problems online or by text, would you prefer to be anonymous, so the other person doesn't know your name or who you are?	A. Yes, definitely<br>B. Yes, but it depends on the situation<br>C. Maybe, I don't know<br>D. No, not necessarily<br>E. No, definitely not<br>F. Prefer not to answer
Q26 (Q32)	Have you ever gone online to anonymously talk to someone one-on-one about your stress or a problem you were having, using a resource like the example shown?	A. Yes, and it was helpful<br>B. Yes, but it was not helpful<br>C. Maybe, I don't know<br>D. No<br>E. Prefer not to answer

Q27 (Q33)	Would you ever go online to anonymously talk to someone one-on-one about your stress or a problem you were having, using a resource like the example shown?	A. Yes, definitely<br>B. Yes, probably<br>C. Maybe, I don't know<br>D. No, probably not<br>E. No, definitely not<br>F. Prefer not to answer
Q28 (Q34)	Would you rather share your stress or problems on social media...	A. That everyone uses, like Instagram or Facebook<br>B. For people with the same stress or problems, like the examples shown<br>C. Not share your stress or problems on social media at all<br>D. Prefer not to answer
Q29 (Q35)	Has the use of social media ever contributed to your stress?	A. Yes, frequently<br>B. Yes, occasionally<br>C. No<br>D. Prefer not to answer
Q30 (Q20)	Have you ever gone online to talk to a counselor or therapist about your stress or a problem you were having?	A. Yes, and it was helpful<br>B. Yes, but it was not helpful<br>C. Maybe, I don't know<br>D. No<br>E. Prefer not to answer
Q31 (Q21)	How comfortable would you be talking to a counselor or therapist using technology, such as video chat or online messaging, like the examples shown?	A. Very comfortable<br>B. Comfortable<br>C. Not sure<br>D. Uncomfortable<br>E. Very uncomfortable<br>F. Prefer not to answer
Q32 (Q22)	Would you be more willing to talk to a counselor or therapist through the use of technology than face-to-face, in person?	A. Yes, definitely<br>B. Yes, probably<br>C. Maybe, I don't know<br>D. No, probably not<br>E. No, definitely not<br>F. Prefer not to answer
Q33 (Q23)	Would you rather talk to a counselor or therapist…	A. Face-to-face, in real life<br>B. In real time using technology<br>C. By exchanging messages back-and-forth online<br>D. Not talk to a counselor or therapist at all<br>E. Prefer not to answer
Q34 (Q24)	Have you ever used a resource like the examples shown to get immediate support in a time of need?	A. Yes, and it was helpful<br>B. Yes, but it was not helpful<br>C. Maybe, I don't know<br>D. No<br>E. Prefer not to answer
Q35 (Q25)	When you need immediate support in a time of need, would you rather…	A. Reach out to someone in your life that can give you support<br>B. Reach out to someone online who has been trained to help people

		C. Wait to talk to a healthcare professional<br>D. None of the above<br>E. Prefer not to answer
Q36	Is there a certain time of day when you tend to experience a high level of stress?	A. During school hours<br>B. Mornings (before school) or afternoons (after school)<br>C. Evenings, nights or weekends<br>D. No, it just depends on the day or situation<br>E. No, I don’t experience a high level of stress<br>F. Prefer not to answer
Q37	Is there a time of day that you prefer to talk to someone about your stress?	A. During school hours<br>B. Mornings (before school) or afternoons (after school)<br>C. Evenings, nights or weekends<br>D. No, it just depends on the day or situation<br>E. No, I don't want to talk to anyone about my stress<br>F. Prefer not to answer
Q38	On a scale of 0 to 10, how would you rate today's Tech to Stress Less event?	0, 10

Note: Schools 3 and 4 had surveys with the alternate order, shown in parentheses in table above
